# Supplementary material for: The Adiponectin Receptor Agonist, ALY688: A Promising Therapeutic for Fibrosis in the Dystrophic Muscle
Source: Cells. 2023 Aug 19;12(16):2101. doi: 10.3390/cells12162101 (PMC10453606; doi:10.3390/cells12162101)
Supplement: Supplementary file 1 [file cells-12-02101-s001.zip › cells-2561477-supplementary.pdf]

Table S1. Antibody Information Chart for Immunohistochemistry and Immunofluorescence

Antibody	Species raised	Dilution	Incubation time	T°	Product code	Source
**Immunohistochemistry**						
IL-1β	rabbit polyclonal	1/200	2h30	RT	ab2105	Abcam, Cambridge, UK
TNFα	rabbit polyclonal	1/100	2h30	RT	ab6671	Abcam, Cambridge, UK
HNE	rabbit polyclonal	1/200	Overnight	4°C	ab46545	Abcam, Cambridge, UK
PRDX3	rabbit polyclonal	1/1000	Overnight	4°C	/	Gift from Bernard Knoops, UCL, Belgium[21]
CD68	rabbit polyclonal	1/500	2h30	RT	ab125212	Abcam, Cambridge, UK
**Immunofluorescence**						
Dystrophin	rabbit polyclonal	1/200	Overnight	4°C	ab15277	Abcam, Cambridge, UK
Laminin-2 (α-2 chain)	rat monoclonal	1/1000	Overnight	4°C	L0663	Sigma Aldrich, Missouri, USA

Table S2. Sequences of real-time PCR primers

Gene	Forward primer (5'-3')	Reverse primer (5'-3')	Melting T°
**Mouse**			
COL1A1	CCGATGGATTCCCGTTCGAGT	GGTGGACATTAGGCGCAGGA	62°C
COL3A1	AAGAGGATCTGAGGGCTCGC	AGGGTGAAAAGCCACCAGACT	62°C
Cyclophilin	AACCCCACCGTGTTCTTC	TGCCTTCTTTCACCTTCCC	62°C
Cyclophilin	TGCAAACAGCTCGAAGGAGACGC	ACGCCACTGTCGCTTTTCGCC	67°C
ERRα	GCCTCTGGCTACCACTACGG	CAGACGCACACCCTCCTTGA	62°C
Mrf4	TGCGGATTTCCTGCGCACCT	GCATCCACGTTTGCTCCTCCTTCC	67°C
Myh1	AGCTTCAAGTTTGGACCCACGGTCG	GCAGCCTCCCCGAAAACGGC	67°C
Myh7	GGTGCCAAGGGCCTGAATGAGGAG	GGTCTGAGGGCTTCACGGGCAC	67°C
**Human**			
AdipoR1	ACTCCTAAGCACCGGCAGAC	CAAGCCAAGTCCCAGGAACA	62°C
IL-1β	GAATCTCCGACCACCACTACA	TGCACATAAGCCTCGTTATCCC	62°C
TBP	CCCCATGACTCCCATGACCC	ACGAAGTGCAATGGTCTTTAGGT	62°C
TNFα	CTCTTCTGCCTGCTGCACTTT	GATGATCTGACTGCCTGGGC	62°C
UTRN	ATTGTAAGGCCCTGAGACGGG	TTCAGGGGCCTCAATTGGCT	62°C

Cyclophilin and *TBP* were used as reference gene in mice/C2C12 and humans, respectively.

Table S3. ELISA kit references

Antibody	ELISA kit number	Source	Previous work using the cited kit in muscle
HNE	ab238538	Abcam	Abou Samra et al, JCSM, 2020
Myh7B	ABIN6968750	Antibodies-online	No citation available
Myogenin	ABIN6957954	Antibodies-online	No citation available
P-AMPK	#7959	Cell Signaling Technology	Abou Samra et al, JCSM, 2020
PGC-1α	MBS707053	MyBioSource	Selvais et al, JCSM, 2023
P-p65	#7173	Cell Signaling Technology	Abou Samra et al, JCSM, 2020
P-RIP	#88918	Cell Signaling Technology	No citation available
P-SMAD2	#7348	Cell Signaling Technology	Dubuisson et al, Front Immunol, 2022
TGF-β	MBS824944	MyBioSource	Narola et al, PLoS One, 2013
TNFα	MBS2884132	MyBioSource	Abou Samra et al, JCSM, 2020
UTRN	ABIN6960407	Antibodies-online	Abou Samra et al, JCSM, 2020

Table S4. Skeletal muscle weight at the end of the study. *Effects of ALY688 treatment on muscle weight*. The total (mg) and relative (corrected by body weight (mg/gBW)) weight of TA, G and Q from the four groups of mice were compared at the age of 12 weeks (sacrifice). Data are means ± SEM; n = 8-10 mice per group. Statistical analysis was performed using one-way ANOVA followed by Tukey's test.
$^{***}P < 0.001$ vs WT.

Muscle	Weight	WT (mean+/-SEM)	mdx (mean+/-SEM)	mdx-T3 (mean+/-SEM)	mdx-T15 (mean+/-SEM)
Tibialis anterior (TA)	Weight (mg)	83.6 ± 1.5	147.8 ± 3.1***	153.5 ± 4.0***	145.7 ± 4.1***
	Relative weight (mg/gBW)	2.9 ± 0.1	4.4 ± 0.1***	4.5 ± 0.1***	4.4 ± 0.1***
Gastrocnemius (G)	Weight (mg)	313.6 ± 2.8	378.5 ± 7.2***	388.9 ± 9.1***	386.6 ± 8.1***
	Relative weight (mg/gBW)	10.8 ± 0.2	11.4 ± 0.1	11.4 ± 0.2	11.6 ± 0.2
Quadriceps (Q)	Weight (mg)	376.2 ± 5.9	568.9 ± 18.3***	569.6 ± 16.2***	563.6 ± 15.2***
	Relative weight (mg/gBW)	13.0 ± 0.3	17.1 ± 0.4***	16.7 ± 0.4***	16.9 ± 0.4***

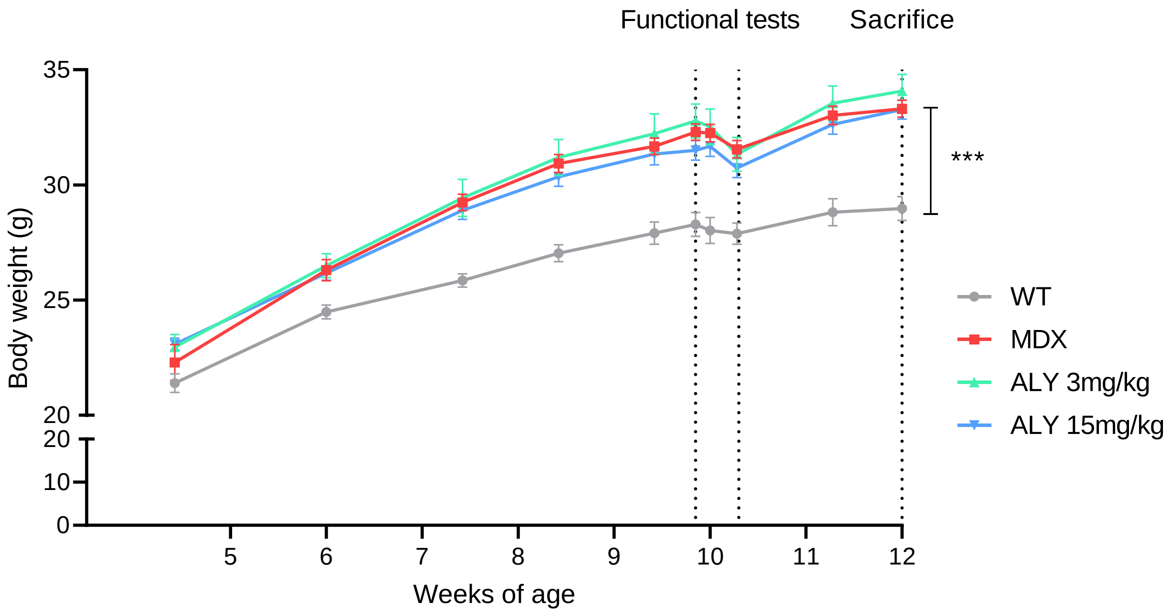

Figure S1. *Effects of ALY688 treatment on body weight*. The body weight of the four groups of mice were compared throughout the study period (from the age of 4 weeks to the age of 12 weeks): WT, mdx (untreated), mdx treated with ALY688 3 mg/kg (mdx-T3) and mdx treated with ALY688 15 mg/kg (mdx-T15) mice. Data are means ± SEM; n = 8-10 mice per group. Statistical analysis was performed using one-way ANOVA followed by Tukey's test. $^{***}P < 0.001$ vs WT. As already described (Coley et al. Hum. Mol. Gen., 2016), mdx mice were bigger than WT ones and their skeletal muscles were heavier, reflecting the well-documented muscle hypertrophy of this model. Most importantly, all the mice displayed harmonious weight growth during the study and no difference was observed between untreated and treated mdx mice, thereby confirming the absence of deleterious effect caused by ALY688

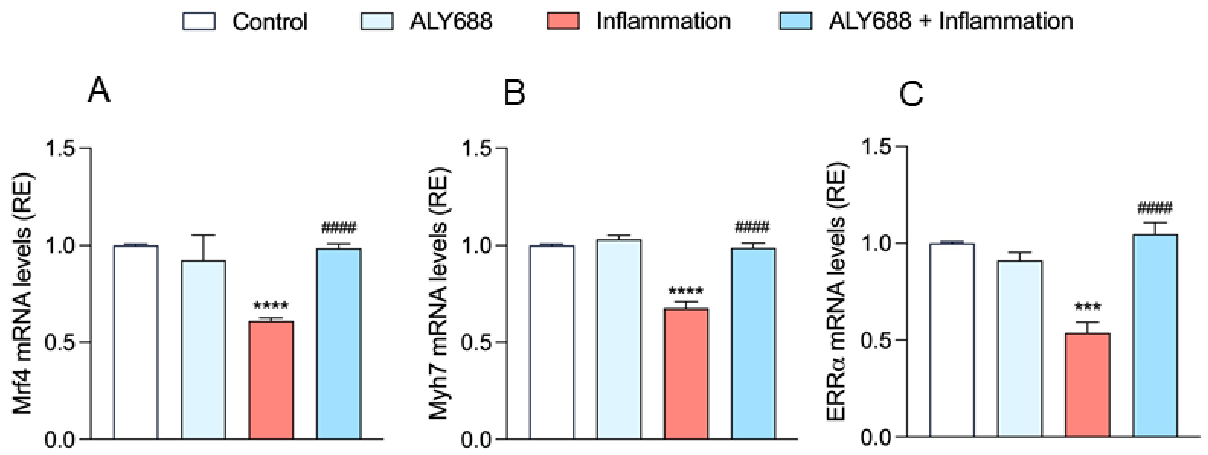

Figure S2. *Effects of ALY688 treatment on muscle markers of differentiation and fibre phenotype in C2C12*. Experiments were performed on murine C2C12 myotubes. Cells were treated or not with ALY688 (100 nM) for 24 h, while being challenged or not with an inflammatory cocktail [TNFα (10 ng/mL) and IFNγ (10 ng/ml)]. **(A-C)** mRNA levels of markers of muscle differentiation (*Mrf4*), oxidative (*Myh7*) fibres as well as of mitochondrial biogenesis (*ERRα*) were quantified. These levels were normalised to *Cyclophilin*. Results were then presented as relative expression compared to control condition (i.e no inflammation, no ALY688). Data are means ± SEM; $n = 4$ independent cultures for all experiments. Statistical analysis was performed using one-way ANOVA followed by Tukey's test. $^{***}P < 0.001$, $^{****}P < 0.0001$ vs controls. $^{\#\#\#\#}P < 0.0001$ vs inflammatory condition.

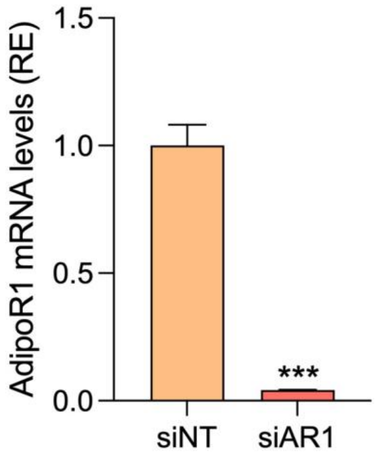

Figure S3. *Effectiveness of siRNA against AdipoR1*. Human myotubes were transfected with siRNA against *AdipoR1* (50 nM) or a negative control [non-targeting, siNT (50 nM)] for 24h. *AdipoR1* mRNA levels were normalised to human *TBP*. The subsequent ratios are presented as relative expression (RE) compared with siNT conditions. Data are means ± SEM for 4 cultures, each obtained from a different donor (i.e., 4 DMD subjects). Statistical analysis was performed using two-tailed paired Student's t-test. $^{***}P < 0.001$ for indicated conditions.

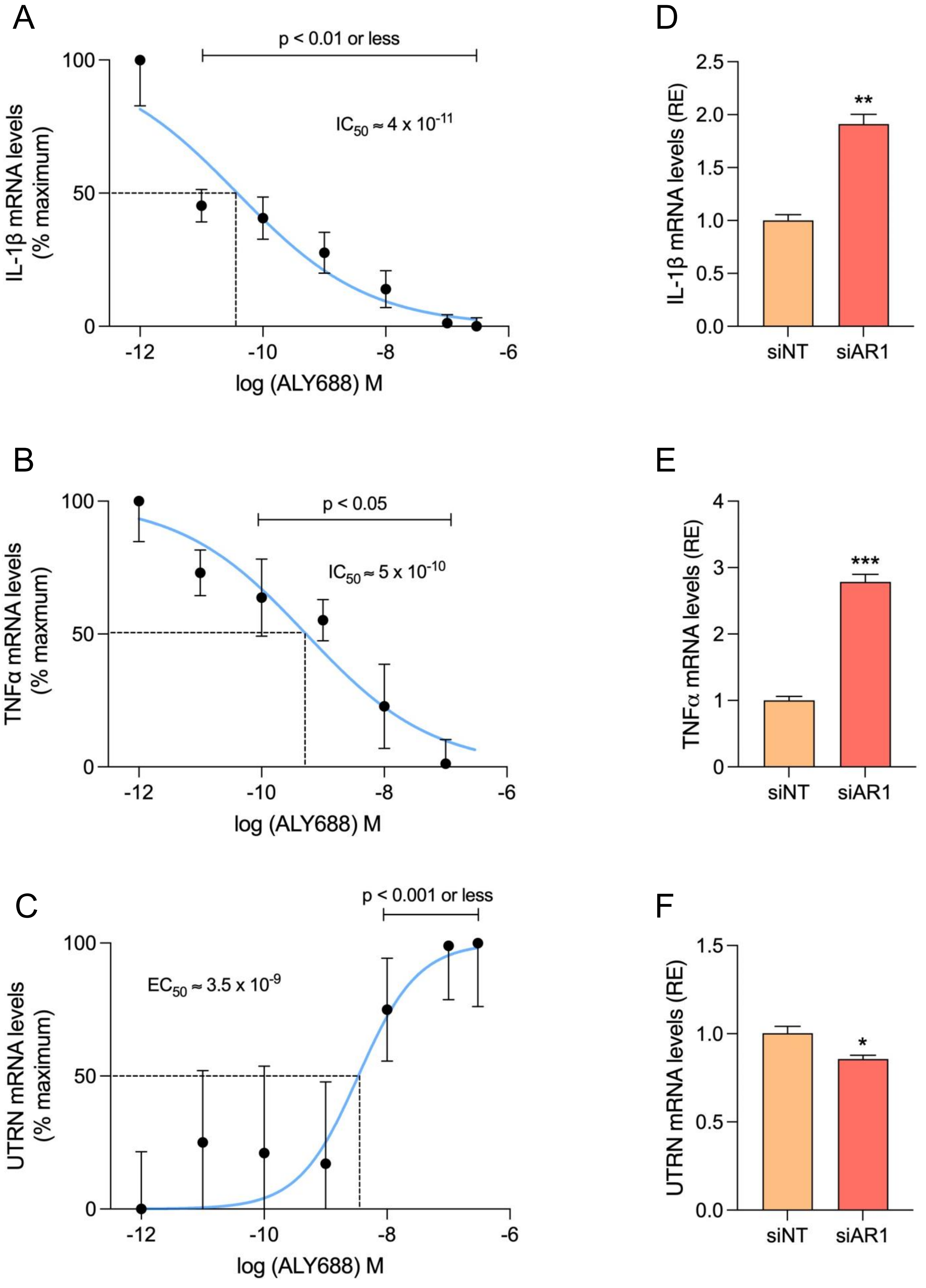
A
p < 0.01 or less
IC50 ≈ 4 x 10-11
IL-1β mRNA levels (% maximum)
log (ALY688) M
B
p < 0.05
IC50 ≈ 5 x 10-10
TNFα mRNA levels (% maxmum)
log (ALY688) M
C
p < 0.001 or less
EC50 ≈ 3.5 x 10-9
UTRN mRNA levels (% maximum)
log (ALY688) M
D
IL-1β mRNA levels (RE)
siNT
siAR1
**
E
TNFα mRNA levels (RE)
siNT
siAR1
***
F
UTRN mRNA levels (RE)
siNT
siAR1
*

Figure S4. *ALY688 recapitulates its anti-inflammatory and pro-UTRN effects in human healthy myotubes, via its action on AdipoR1*. **(A-C)** Dose-response curves illustrating the effects of ALY688 on *IL-1β*, *TNFα*, and *UTRN* mRNA levels in primary cultures of myotubes obtained from healthy subjects. Cells were treated or not with several concentrations of ALY688 (from 10 pM to 300 nM) for 24 h, while being challenged with an inflammatory cocktail (human recombinant TNFα/INFγ, each at 15 ng/mL). mRNA levels were normalised to human *TBP*. Data were then presented as % of the maximal levels obtained either without (A, B) or with 300 nM ALY688 (C). **(D-F)** In some experiments, cells were first transfected with siRNA against AdipoR1 (50 nM) or a negative control [non-targeting, siNT (50 nM)] for 24h and then treated with ALY688 (100 nM) combined to inflammation (TNFα/IFNγ) for an additional 24h. After normalisation, mRNA levels were presented as relative expression (RE) to siNT conditions (D-F). Data are means ± SEM for 4 cultures, each obtained from a different donor (i.e., 4 healthy subjects). Statistical analysis was performed using repeated measures of ANOVA followed by Dunnett's test (A-C) or two-tailed paired Student's t-test (D-F). $^{*}P < 0.05$, $^{**}P < 0.01$, $^{***}P < 0.001$ vs siNT.

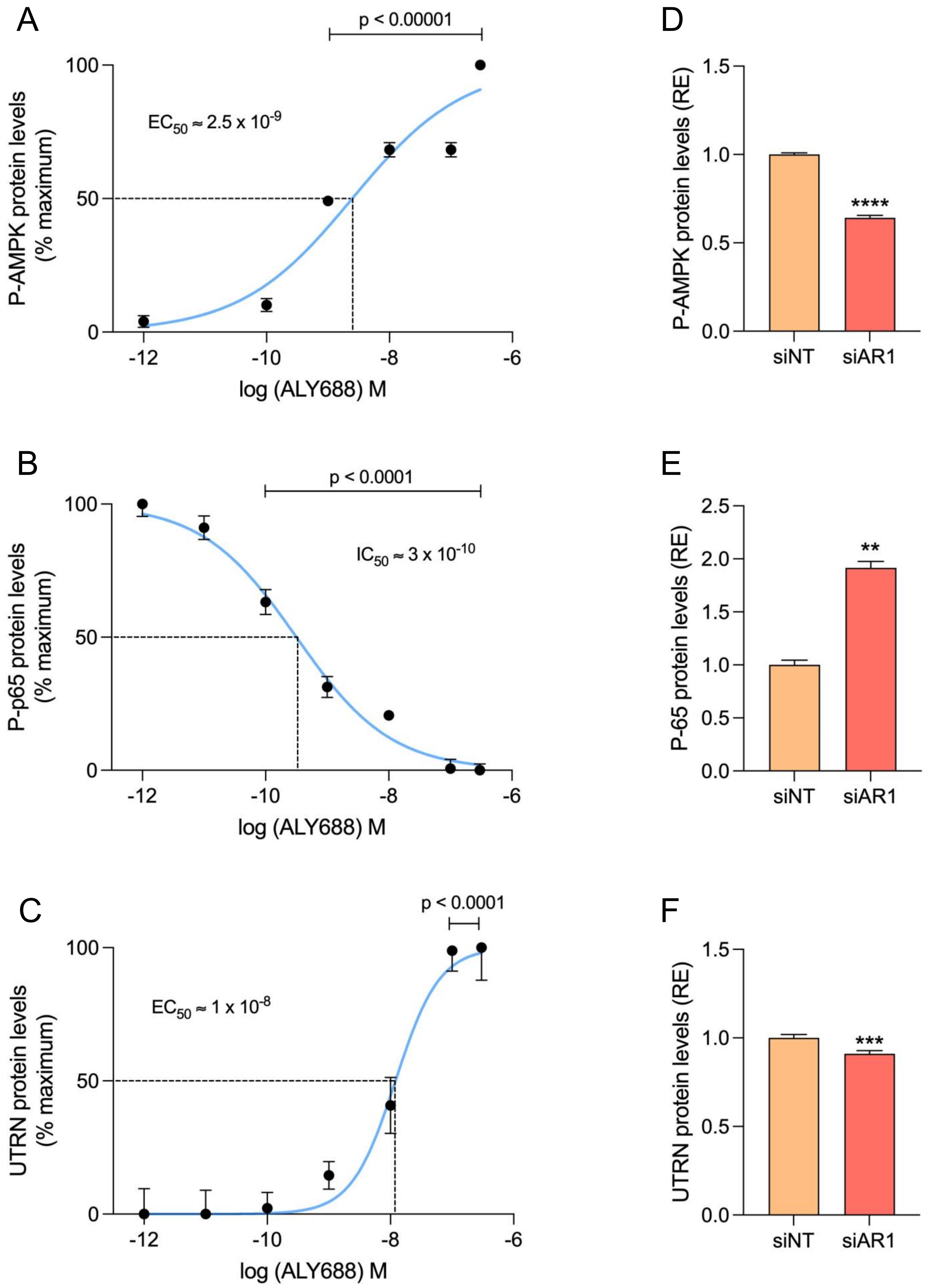
A
p < 0.00001
EC50 ≈ 2.5 x 10-9
P-AMPK protein levels (% maximum)
log (ALY688) M
B
p < 0.0001
IC50 ≈ 3 x 10-10
P-p65 protein levels (% maximum)
log (ALY688) M
C
p < 0.0001
EC50 ≈ 1 x 10-8
UTRN protein levels (% maximum)
log (ALY688) M
D
P-AMPK protein levels (RE)
****
siNT
siAR1
E
P-65 protein levels (RE)
**
siNT
siAR1
F
UTRN protein levels (RE)
***
siNT
siAR1

Figure S5. *ALY688 treatment recapitulates its effects on key effectors of the AMPK signalling in human healthy myotubes, via its action on AdipoR1*. **(A-C)** Dose-response curves illustrating the effects of ALY688 on AMPK and NF-κB activity (P-p65 subunit), and UTRN protein levels in primary cultures of myotubes obtained from healthy subjects. Cells were treated or not with several concentrations of ALY688 (from10 pM to 300 nM) for 24 h, while being challenged with an inflammatory cocktail (human recombinant TNFα/INFγ, each at 15 ng/mL). Levels of each protein were measured by ELISAs and then presented as % of the maximum achieved either without (B) or with 300 nM ALY688 (A, C). **(D-F)** In some experiments, cells were first transfected with siRNA against AdipoR1 (50 nM) or a negative control [non-targeting, siNT (50 nM)] for 24h and then treated with ALY688 (100 nM) combined to inflammation (TNFα/IFNγ) for an additional 24h. For each protein, levels were presented as relative expression (RE) compared with siNT conditions. Data are means ± SEM for 4 cultures, each obtained from a different donor (i.e., 4 healthy subjects). Statistical analysis was performed using repeated measures of ANOVA followed by Dunnett's test (A-C) or using two-tailed paired Student's t-test (D-F). $^{**}P < 0.01$, $^{***}P < 0.001$, $^{****}P < 0.0001$ vs siNT.
